# Hospital and Institutional News

**Published:** 1912-01-20

**Authors:** 


### HOSPITAL AND INSTITUTIONAL NEWS.

### THE VOLUNTARY HOSPITALS AND THE INSURANCE ACT.

Parliament meets next month, and it is time that those responsible for the administration of our voluntary hospitals should at once join hands and determine the policy which they will pursue in regard to the Insurance Act. Apart from London a general feeling of alarm has spread, and almost everywhere evidence is forthcoming that the Insurance Act may adversely affect the revenue of many voluntary hospitals. In London opinion is divided, but those hospitals which have to raise a considerable income, as Mr. Alvey, the able secretary of Charing Cross Hospital, has made perfectly clear, will suffer most. Hence he is in sympathy with the views held by voluntary hospital managers in the provinces and Scotland. It may be difficult no doubt to arrive at some basis acceptable to each of the voluntary hospitals, but the plan for amending the Act, which we refer to later, whilst conserving the voluntary principle of hospitals in this country, would leave each such hospital free to enter into an agreement with the Insurance Commissioners, or not, as its managers might decide to be best. After all, hospital managers will no doubt support the plan best calculated to further the interests of the institution for whose administration they are responsible.

### THE PREVAILING VIEW ON THE ACT.

The view then is now rapidly spreading throughout the hospital world that voluntary hospitals may be seriously injured by the new Act. It should be practically certain that the out-patient department as it exists to-day will disappear, to be replaced by a consultation branch of hospital work in co-operation with the whole body of medical practitioners in the district which each hospital serves. Over the doorways of the new consultation and reorganised casualty departments might appear the words: "Out-patient system ended but amended." This suggestion is made in answer to a request in a contemporary for a suitable motto. The reorganised casualty department will have to be worked on business lines with an ample number of assistants, so that the sifting of all applicants for relief which must be done here may be reduced to the simplest possible proportions. Should our forecast as to out-patients prove generally correct, it should result in blessings to the patients and to the hospitals, and give great satisfaction to the medical profession and the public.

### NOBLESSE OBLIGE.

The *Medical Press*, under this heading, referring to the past indiscriminate distribution of free hospital treatment, properly declares that skilled in-patient treatment, both surgical and medical, is every year becoming a more complex and costly affair. The writer points out that under the new Act there will be precisely the same need of in-patient treatment for serious cases as there has ever been, and then appeals to us to devise some way out of the wood, so far as the indoor treatment of such cases is concerned. Our contemporary cannot see, apparently, that the breadwinner who was unable, before the passing of the National Insurance Act, to provide for himself and his family when ill, and was so entitled to free hospital treatment, will be ineligible for free in-patient relief in a voluntary hospital as an insured person, for he is then to be provided with adequate medical treatment under the Act, and thus freed from his former dependence upon charitable relief. We venture to ask the writer to turn this fact over in his mind, and to look at it from every point of view, when we are pretty confident he will soon realise that an insured person cannot, without grave abuse, be eligible for free in-patient treatment in a voluntary hospital. As to a wage or income limit, that is already in force in certain hospitals, and is rigorously applied in the case of great hospitals in the United States, where no patient receives free in-patient relief unless he can prove to the satisfaction of the superintendent that he is entitled to it on the merits. Failing such proof he is relegated to a paying bed, for which he gives a daily contribution according to his circumstances and means.

### NATIONAL INSURANCE AND THE PAY SYSTEM.

The Insurance Act should prove a stepping-stone to the creation of a perfected system of national health provision. It should remove a multitude of hardships which weigh grievously upon the happiness and welfare of thousands of people whose means are relatively small, and who belong to the least successful and lower-paid members of most professions and the salaried classes generally. For these often highly educated and most respectable people there is really little or no hospital provision in this country at the present time. Their needs might readily be supplied by the adoption of the Continental and American plan of classifying the wards or sections of the hospitals, so as to secure provision for (1) those who can pay a proportion, at any rate, of the cost of their residence in a hospital; (2) those who can pay the whole cost of such residence; and (3) those who can provide not only the cost of their maintenance, but of their medical and other treatment. We showed very clearly how to prepare for a perfected hospital and health service for Great Britain in our issue of December 9. Those who are interested in this question of providing a perfected hospital system should obtain a copy of the pamphlet published by The Scientific Press, Limited, entitled, "The National Insurance Act and Hospital Benefit," together with that on National Insurance and the Voluntary Hospitals. The pamphlet relating to hospital benefit just mentioned will make it clear to any reader how simple a thing it is readily to provide that the scheme of National Insurance shall result in a perfected hospital system, whilst it will perpetuate the Voluntary Hospitals in a strengthened and extended form.

### CAN THE NATIONAL INSURANCE ACT BE AMENDED.

Not only can the National Insurance Act be amended, but the Commissioners have had to recognise that it must be amended before they can carry out the Act as it stands to-day. Indeed we have it on good authority that at least one Commissioner has admitted that it will require not one but probably four amending Acts before the present scheme of national insurance as it stands can be made workable and satisfactory. Seeing therefore that an amending Act, no matter what other legislation the Government may desire to further, must be passed through the House of Commons in the ensuing session of Parliament, it is of the first importance that the whole of the voluntary hospitals shall combine to secure that any such Act shall include power to provide hospital benefit on a *pro rata* basis through the voluntary hospitals. How simple the necessary amendments to the existing Act are, and how readily, economically, and practically it is possible to provide in the amending Act for a perfected hospital system, will be found fully explained in the pamphlet entitled "The National Insurance Act and Hospital Benefit" published by the Scientific Press, which we commend especially to our contemporary the *Medical Press*, who has pointedly asked us to supply information upon the matters we have dealt with in this and the preceding paragraphs. All that is needed is that the hospitals should awake and act now before Parliament meets, and not afterwards. No doubt the Commissioners or the representatives of the Government are preparing, if they have not already prepared, a short amending Act to enable the present one to be put into efficient action. So time is of the essence of success, and the managers of no voluntary hospital need hesitate to co-operate, for the reason that the amendments contemplated will leave it free to every voluntary hospital to make, or refuse to make, an agreement with the Insurance Commissioners for the admission of insured cases as in-patients. It will therefore be optional on their part to enlarge or decline to enlarge the area of their work by accepting Government inspection, and so to be placed in a position to perfect the voluntary hospital by extending its usefulness to all classes of the community who may need in-patient hospital care.

### LIVERPOOL'S NEW HOSPITAL PRESIDENT.

Mr. Thomas Woodsend, who has been unanimously appointed to the presidency of the general committee of the Royal Southern Hospital, Liverpool, succeeds Mr. John Rankin, who has held that office provisionally since the death of Mr. William Adamson. Mr. Woodsend is a member of the merchant firm of Duncan Fox and Co. Mr. Woodsend joined the committee of the Royal Southern Hospital in July 1906, and from May 1908 to December 1909 also served on the Board of Economy. It should be recalled that Mr. John Rankin acted provisionally as President from September 5, 1911 until January 8, 1912. His presidency marks the largest collection at the annual church service, held on November 27, 1911, which was attended by the Right Hon. Lord Derby, Lord Mayor, and the Lady Mayoress, who also paid an official visit to the hospital after the service. On this occasion the amount collected was £270 7s. These services commenced in 1843. The Committee is exceedingly glad that Mr. Rankin's association with the Hospital, although time would not allow of him holding the office permanently, will not be lost as he will still be a vice-president.

### LATEST RETURNS OF THE SATURDAY FUND.

It is interesting to observe that over ten thousand pounds were remitted to the Hospital Saturday Fund within eight days of its closing date, namely, the 8th ult., and on the final day alone, we understand, about £6,000 was received. The total collections made on the Fund's behalf in the business houses and workshops of London in 1911 amounted to £37,000 odd, a gratifying increase of almost £2,500 over the sum collected in the previous year. We can only hope that this advance may be maintained this year, but the outlook for the voluntary support of hospitals, as the example we quote from Norwich clearly shows, is undoubtedly serious.

### THE LATE MR. LABOUCHERE AND VOLUNTARY CHARITY.

The death of Mr. Labouchere, which we regret for his many excellent qualities, is interesting to the hospital world. For it removes a gentleman who defended the cause of true voluntary charity by his scathing exposure of bogus institutions and individuals in the caustic paragraphs of "Truth." On one occasion, it is true, he fell foul of a skin hospital, the financial management of which was asserted to be unsatisfactory, but in the end the case was settled; another periodical, by the way, less adroit than "Truth" having to pay £300 damages. This was characteristic of the skill and seriousness with which "Truth" has always prosecuted its vigilance work. Personally Mr. Labouchere was one of the most charming and delightful of companions. We shall never forget the profit and pleasure of a journey we once took in his company from Verona to Florence. Mr. Labouchere leaves his paper excellently managed and edited, under the direction of Mr. R. A. Bennett, who has been Mr. Labouchere's right hand for many years. The continued excellence and success of "Truth" is therefore assured.

### A LINCOLN HOSPITAL'S ECONOMIES.

Mr. Arthur Moore, Secretary of the Lincoln County Hospital, had a very pleasant task last week in explaining how the present balance of nearly one thousand pounds has been obtained. Oddly enough new machinery seems to have been an important factor in this result, and great credit must be given to the engineer and his department, which by a new hot-water apparatus has saved £50 in the cost of water, while £77 has been saved on steam coal. By the way Mr. C. C. Sibthorpe is again treasurer, and the weekly board has a new member in the person of Canon Bell, who thus fills the vacancy caused by the death of Mr. Jekyll. It is also interesting to add that until a salary of £100 was offered for a junior house surgeon no applicants were forthcoming; the moment that figure was advertised, in place of the £75 which preceded it, nine candidates appeared.

### THE MEDICAL TRAINING OF MISSIONARIES.

With reference to our remarks a month ago on the imperative need for training would-be missionaries in medicine, Dr. Edwin A. Neatby, M.D., writes to remind us of the existence of the London Missionary School of Medicine, which has its head-quarters in the Homœopathic Hospital, Great Ormond Street. The school was founded in 1903, and its prospectus states that the methods of practice taught are not confined to homœopathy, and further that "its students do not pose as medical missionaries. Each one is bound by a written undertaking not to assume the position or title of a qualified medical missionary at home or abroad." The whole course lasts three college terms. Clinical practice in the wards and out-patient department is arranged to suit the needs of the students. Anything which tends to impress upon religious bodies the overwhelming necessity for all missionaries to have at least an elementary knowledge of medicine is to be welcomed, and we hope that Livingstone College and the London Missionary School of Medicine definitely will work for the day when no one will be allowed to offer himself as a candidate for the mission field unless he can show some certificate of proficiency in medicine. That, and that alone, is the goal worth working for.

### NEW APPOINTMENTS AT BOLTON INFIRMARY.

A third house surgeon is to be appointed at the Bolton Infirmary, the matter being now in the hands of the medical committee. Another change is the appointment of the Rev. Samuel Russell to the general committee, who fills the place previously occupied by the late Rev. T. B. Harrowell.

### A SCHOOL CLINIC FOR PORTSMOUTH.

Apparently Portsmouth is to follow the example of Bradford and to have a school clinic of its own. Such at least is the recommendation of Mr. J. Dummer, a member of the Portsmouth Education Committee, which followed his advice the other day in recommending the establishment of a school clinic to the full committee. Mr. Dummer pointed out that it was little use indicating physical defects in children unless these defects were followed up. Probably the local medical men will be asked to submit a list of names, from which four will be appointed to attend the children. It is also suggested that Dr. Inman, the oculist at the Eye Infirmary, should be appointed "eye specialist" at £1,000 a year. If the scheme is quickly taken up it is possible that the clinic may be in working order in three months' time.

### AN INFIRMARY BOYCOTTED BY DOCTORS.

A storm of opposition has been created by the Brighton Board of Guardians' recent proposal to appoint a consulting and operating surgeon at an honorarium of fifty guineas a year. The local practitioners are so indignant at the smallness of the sum offered that the local division of the British Medical Association not merely passed a resolution "disapproving" of any application for the post unless the salary be at least one hundred guineas but has published a general warning. The Guardians therefore are considering the matter, and their decision no doubt will depend partly on precedent, and partly perhaps on their knowledge of the results that usually attend the importation of "a blackleg" from another district.

### DISASTER CAUSED BY THE INSURANCE ACT AT NORWICH.

Mr. R. E. Horsfall has probably taken the wisest course in explaining to the public the disastrous effect which the Insurance Act has had on the funds of the Norfolk and Norwich Hospital. Mr. Horsfall went so far as to say that though the year was barely a fortnight old, they had had a large number of subscriptions discontinued and reduced, many subscribers writing to say that it was on account of the Insurance Act. The only conclusion he could draw was that if the Act were enforced as it at present stood, and if the hospital were carried on on its present lines, it would be bankrupt in less than five years. Some people had a vague idea that voluntary hospitals would be compensated for any falling off in funds by being relieved of some of their obligations. What the nature and value of those obligations were he had never seen stated, and he had his doubts whether they would equal the loss of income that would occur when subscribers realised that they were compelled to pay far more for the medical relief of the poor classes than they had ever given voluntarily. Whether this would be the beginning of the end of voluntary hospitals remained to be seen. From this it will appear that the problem before the board and the secretary, Mr. Frank G. Hazell, is serious in the extreme. We trust that the effect of this announcement will be to awaken the East of England to the danger with which the voluntary system is threatened, and credit is due to Mr. Horsfall for his outspoken remarks, which already have created a deep impression, that has extended far beyond Norwich.

### SCOTLAND'S MEMORIAL TO KING EDWARD.

The endowment of phthisical research is the scientific fashion of the moment, and probably it has come as no great surprise to anyone that Scotland has decided to commemorate the late King by the establishment and endowment of an Institute of Preventive Medicine to be called after him. This decision was arrived at as the result of a conference of Scottish local authorities which was held last week at Edinburgh. Needless to add, the Institute primarily is to be dedicated "to research in relation to tuberculosis," and as if to make quite apparent Scotland's orthodoxy on this matter, a committee also has been appointed to consider the question of building and maintaining sanatoria. With a similar motive in Wales, and with the Chancellor's amorphous "sanatoria benefit," tuberculosis research has come now to the full tide of its popularity. It is no business of ours to minimise the evils of phthisis, or to criticise the immense amount of research now being undertaken in its name. We merely think that for the moment enough public money has been spent on this branch of preventive medicine, and think it worth while to mention that we should find no difficulty in mentioning six other subjects equally in need of money for research. There is sometimes a sad lack of scientific imagination in the allocation of memorial funds. Tuberculosis research, at any rate, is as well provided for at present as its most earnest disciple could wish.

### LATEST PHASE OF THE DILKE MEMORIAL.

It is to be noted that the memorial scheme by which a hospital for the Forest of Dean was to be built in memory of Sir Charles Dilke has, like certain other similar schemes, been suffering from divided counsels. The free hospital, however, was the original proposal, and moreover has been long wished for, so we are particularly glad to notice Sir William Wedderburn's intervention at the present slightly critical stage. Sir William makes the important announcement that an anonymous admirer of Sir Charles Dilke, rather than see the proposed hospital abandoned, has come forward and promised to "give half the cost of such a hospital, if the other half is provided by subscription." The whole situation is thus changed, and Sir William Wedderburn and his anonymous friend are to be congratulated on the timeous moment of their appearance. Local public spirit now has what is probably a unique chance of fulfilling a perfectly natural, but hitherto almost impossible, local ambition.

### FOUNDER'S GIFT TO A HOSPITAL FOR INCURABLES.

Mrs. William Wilson, well known in Harrogate as the lady who in 1881 gave the Yorkshire Home for Incurables in memory of her daughter, Miss Marjorie Wilson, has just presented the Home with a new recreation-room. The chairman of the Board of Management, Mr. H. Milling, presided at the presentation, and in proof of the Rev. A. Freeman's words that the new room would increase the patients' opportunities for enjoyment, a concert was given in the evening. Mr. and Mrs. J. S. Rowntree, the mayor and mayoress of Harrogate, were present, and the secretary, Mr. James Hindell, may be congratulated on the addition to his institution.

### A LADY AS HOSPITAL SECRETARY.

The new operating-theatre which was opened a few days ago at S. Barnabas Cottage Hospital, Saltash, Cornwall, has cost £800, of which, we are sorry to learn, some £600 has still to be raised. The committee, Mr. F. Gover, Mr. P. S. Boger, and Mr. E. W. Rashleigh, did not, we believe, press this home at the opening, and except for the sale of work which followed the speeches, to the mayor, Mr. E. Smith, belongs the credit of promising £5, the only promise apparently made at all. It should be remembered that this Cottage Hospital, which Mrs. Caroline Ley founded in 1889 in memory of her husband, is under the care of the Sisters of S. Margaret's, East Grinstead, together with that of the local committee referred to. It is a notable fact about this hospital that the work of the honorary secretary and treasurer is in the hands of the sister-in-charge. We might recall there are other hospitals where the secretary is a woman. We think it would be unfortunate if the neighbourhood did not make itself responsible for the cost of the improvements, which include an ironing-room, as the success of any voluntary hospital largely depends upon intelligent local interest.

### THE MOVE FOR SCHOOL CLINICS AT CARDIFF.

Exceptional interest was given to the last ordinary meeting of the board of King Edward VII. Hospital, Cardiff, by a resolution of the medical staff urging, in effect, the city to consider the advisability of establishing school clinics. It was contended, especially by Dr. Paterson, who is an enthusiastic believer in these establishments, that only by treating the less serious cases at clinics can the hospital's out-patient department be relieved from the congestion which the present number of children causes to the disadvantage of more urgent cases. Eventually it was decided, thanks to Mr. L. Samuel's motion, which was seconded by Mr. W. Thomas, that a committee of the board should meet the medical board to discuss the matter further. From the recent discussions on it, and from the important move in this direction at Portsmouth, it is clear that our series of articles on "The School Clinic" could not have appeared at a more fortunate time. They have set forth from every point of view those facts on the recognition of which the success of the school clinic entirely depends.

### MANCHESTER ROYAL INFIRMARY IN THE COURTS.

A very interesting judgment was delivered the other day by Mr. Jelf Petit at the Denbighshire Quarter Sessions in an appeal of the Manchester Royal Infirmary against the assessment of certain premises, including its Convalescent Home at Colwyn. The question raised by the respondents, the Conway Union, was that the cost of construction must be the measure by which the amount of assessment should be aimed at. But, said Mr. Petit, it was quite impossible to apply this principle universally. In this instance there had been extra cost in order not to destroy the amenities of the locality, and he thought it would not be fair to take this extra cost into account for the purposes of the assessment. Again, it was sometimes found that local by-laws necessitated a considerable expenditure on institutions of this kind if conformity was insisted upon. The Manchester Royal Infirmary urged, therefore, that the original cost of construction must be entirely left out, and that the Court was concerned only with what rent a hypothetical tenant would consent to pay. He could not adopt this view either. The original cost of the premises must be taken as a starting-point on which to base the calculation, and other evidence must be taken into consideration to guide them in arriving at a fair assessment.

### THE VERDICT AND THE MORAL.

The upshot was that the assessment on the Infirmary's premises at Old Glanydon was upheld, while that in respect of New Glanydon was reduced from £640 net to £436 net. The Infirmary, moreover, was given costs in the appeal, excluding such part of its costs as related solely to the Old Glanydon premises. We have given this judgment at some length because anything which tends to throw light on the obscure subject of rating is of importance to institutions, but it will be generally regretted that this case has not established, or at least projected, a reasonable principle for future reference. Two principles have been rejected, but none put in their place. It is the same story, a typical example of the genius of English law, the absence of code or principle, and the deciding of each case on its merits: for that is what "taking other evidence into consideration" really means.

### THE SHORTEST-LIVED PROFESSION.

We believe we are correct in stating that medicine is one of the shortest-lived professions, that its members, in other words, die earlier than is the case in most other occupations. Medical centenarians are therefore phenomena more interesting, because rarer, than centenarians in general. Essex boasts one at the moment in the person of Mr. Edgar Jones, M.R.C.S., who attained his hundred-and-third birthday last week. His family appears to show a predisposition to longevity; Mr. Jones' father, it is stated, having lived to the age of ninety-two, and two brothers and one sister having all passed the age of ninety.

### FRIMLEY SANATORIUM AND THE FARNHAM GUARDIANS.

A rather curious case arose lately in connection with the Frimley Sanatorium and the Farnham Guardians. The Frimley authorities requested the Guardians to remove to the infirmary an advanced case which had been sent to Frimley and paid for by the Barnsley Union. The Sanatorium, which is for incipient cases, wished to be rid of this case, and, as the Barnsley Union, it is said, "opposed facilities" for his return, called on the relieving officer, who, on the ground that the patient was not destitute, refused to remove him. Apparently the matter has been mentioned to the Local Government Board, a high official of which is stated to be on the Committee of the Sanatorium. A Local Government Board inspector then advised the clerk that, if the Sanatorium handed over the patient, the Guardians would be responsible for his relief. The question is not so much whether a Union's guarantee to pay 25s. a week for a patient prevents him from being technically destitute, as whether a sanatorium can refuse its responsibility when having once accepted what has proved to be an unsuitable case. In any event the Guardians have not given way, and the patient is too ill to be moved.

### RESIGNATIONS FROM A HOSPITAL STAFF.

Mr. W. J. R. Fox has resigned the presidency of the Batley and District Hospital, and his resignation will probably take effect at the end of the month. Dr. R. A. Forsyth, M.D. Glas., who has been on the medical staff for twenty-five years, has also resigned.

### A ROMANTIC BEQUEST TO A HOSPITAL.

The late Mr. John Littlehales, of Warwick, licensed victualler, will be remembered, locally at any rate, as a testator whose will contained a romantic provision, which will condition for seven years the bequest of one thousand pounds which, subject to that, he has left to the Birmingham General Hospital. The testator had a nephew, Mr. Robert Littlehales, who left England some years ago, and to him the thousand pounds is to be given—if he can be found within seven years of his uncle's death. After that, the money is to revert to the hospital. It is not suggested, by the way, that while the search for the errant nephew is being undertaken the interest on the thousand pounds should be given as a solatium to the hospital. Hospital bequests not rarely depend, as in this case, on some romantic contingency, and the subject of bequests with romantic conditions is one that would produce an interesting result, as a sidelight on hospital history, if pursued by any secretary in search of a hobby for his leisure hours.

### BRISTOL'S HOSPITAL SUNDAY: ITS CHANCE THIS YEAR.

The date fixed for collections in aid of the Lord Mayor of Bristol's Hospital Sunday Fund is January 28. For many years past Highbury Congregational Church, where the present Lord Mayor of Bristol, Mr. Frank W. Wills, has attended from childhood, has been to the fore in this matter. We hope, therefore, that his appeal to the clergy for a record collection will inspire first his own church and then others, so that Bristol may have the double honour of being not merely a stronghold of Hospital Sunday, but a stronghold unaffected by the set-back which through local inertia the Sunday Fund movement has suffered lately in various parts of the country.

### FURTHER EXTENSIONS AT IPSWICH HOSPITAL.

Mr. Herbert W. Mason outlined last Saturday a scheme for the extension of the Nurses' Home at the East Suffolk and Ipswich Hospital by the addition of a block containing sixteen bedrooms, bathrooms, store-rooms, and a nurses' study. The original building has been designed with a view to future extensions, and the time has now arrived for these to be made. The reason is that during the year the accommodation for patients has been increased by some sixteen beds as a result of the gift of open-air balconies to the children's wards by Mr. J. D. Cobbold. These balconies, it will be remembered, were constructed to allow of the permanent use of all beds placed therein in all weathers. These additional beds naturally have necessitated an increase in the nursing staff, the accommodation for whom is already limited. In order to provide the architect's estimate of £2,500 a proposition that the money should be taken out of invested capital was carried unanimously, and the scheme of extension adopted. Further developments, moreover, are anticipated, for Mr. F. Messent, Chairman of the Board of Management, foreshadowed large extensions of the $x$-ray department in the near future.

This capital expenditure has been sanctioned on the ground that it would be inopportune to make a public appeal, partly for general reasons and partly because little more than a year has elapsed since the previous extension scheme was carried out.

### A PATIENT'S MEMORIES OF OPERATIONS IN 1847.

In a past number of the *University College Hospital Magazine* a most interesting account was given of the two famous operations performed there by Robert Liston in 1847, the first in England under ether anæsthesia. A sequel to the narrative of that historic day is now published in the same magazine, being no less than an interview with a patient who underwent in University College Hospital amputation through the thigh earlier in the same year, and therefore without the benefit of anæsthesia. This patient must surely be about the only survivor of Liston's amputation cases, for the great surgeon died only a few months later; and there can also be few indeed who can say they had so severe an operation without the benefit of either antiseptic surgery or anæsthetics. The patient was at that time thirteen years of age, and was suffering from what appears to have been fairly advanced tuberculosis of the knee joint. Liston advised amputation; and when the boy asked if it would hurt, replied "Not any more than having a tooth out." The amputation was accordingly performed; and the patient testifies that it really did hurt not nearly so much as he expected, in fact hardly at all. The operation was completed in twenty seconds! and the boy refused at first to believe that the leg was off. It has, of course, to be remembered that the sewing up was not performed in those days until several hours after the actual operation; and in this particular case it is recorded that this part of the operation hurt far more than the amputation itself. Truly patients, as well as surgeons, had to be cast in a somewhat heroic mould in 1847 and previously.

### AN AUSTRALIAN CHAIRMAN'S HOSPITAL TOUR.

The Chairman of the Royal Prince Alfred Hospital, Sydney, Professor T. P. Anderson Stuart, is leaving for an extended tour in England and on the Continent, during which he will devote much time to investigating the organisation and conduct of hospitals. Professor Anderson Stuart, who is by the way Dean of the Faculty of Medicine and Professor of Physiology at Sydney University, is a very keen hospital chairman and deserves much of the credit that has been brought to his hospital owing to the establishment of the fine pathological department which has been added of recent years. Hospital managers over here will look forward to seeing something of an Australian confrère, for intercourse between the hospitals throughout the Empire is as scanty as it is frequent in England itself. The British Hospitals Association, doubtless, will be able to supply any information that might be required to ensure the success of at least the English section of Professor Anderson Stuart's tour.

### HOSPITAL SUNDAY AND THE CHURCHES.

The threatened decadence of Hospital Sunday, owing to the supineness of many of the clergy of all denominations and sometimes to that of the hospital chaplains themselves, makes the following analysis of the contributions of all religious bodies towards the London Sunday Fund of interest to all hospital workers. For it we are indebted to the *National Church*, in which it is an annual feature:—

| | |
|---|---|
| Church of England | £28,921 |
| Congregationalists | 1,704 |
| Jews | 1,452 |
| Presbyterians | 1,068 |
| Wesleyans | 826 |
| Roman Catholics | 710 |
| Baptists | 644 |
| Unitarians | 404 |
| Church of Scotland | 327 |
| Theistic Church | 200 |
| German Lutherans | 97 |
| Catholic Apostolic | 75 |
| Greek Church | 74 |
| Methodists (Primitive) | 71 |
| Society of Friends | 64 |
| Foreign Protestants | 47 |
| Methodists (Welsh Calvinistic) | 45 |
| Methodists (United) | 19 |
| Reformed Episcopal Church | 13 |
| Swedenborgians | 11 |
| Free Church of England | 11 |
| Moravians | 4 |
| Various | 248 |
| Total | £37,040 |

This total, which omits the odd shillings, is £3,710 less than the sum raised in 1910.

### AN ERROR CORRECTED.

We much regret the printer's error which crept inadvertently into Dr. Alfred Norman's article on "Electricity in Modern Medicine" last week. On page 381 the second line above "fig. 2" should read "Fig. 2 illustrates a useful type of hand lamp." In place of this the third line above that was repeated, by a printer's error for which we are sincerely sorry.

### THIS WEEK'S DRUG MARKET.

Business in the drug market is in full swing again, and while in volume it cannot be considered large, it is satisfactory as compared with the amount usually done at the beginning of a new year. Changes in value are few in number, and, on the whole, of an unimportant character. Opium and its alkaloids are unchanged in value, and there are no signs that prices are likely to give way; on the contrary, there is a probability of a further advance. Glycerin is also unchanged in price, but a further reduction is not altogether improbable. Last season's cod-liver oil still tends lower in value, and buyers are not likely to come forward until reports are to hand concerning the Norwegian fishing, which should begin in about a week's time. Borax and boric acid have been slightly advanced in price. Carbolic acid is still tending upward in value. A further advance in the price of santonin has taken place. Oil of star aniseed is tending dearer. At the recent public drug sales in Mincing Lane, ipecacuanha sold at lower prices, and buchu leaves tended dearer.

